# Sulfoquinovosyl diacylglycerol, a component of Holy Basil *Ocimum tenuiflorum*, inhibits the activity of the SARS-CoV-2 main protease and viral replication *in vitro*

**DOI:** 10.1007/s11418-024-01855-6

**Published:** 2024-11-25

**Authors:** Hinako Koze, Masayuki Sudoh, Satoaki Onitsuka, Hiroaki Okamura, Takeshi Ishikawa, Fumito Tani, Yukako Miyata-Yabuki, Mikako Shirouzu, Masanori Baba, Mika Okamoto, Toshiyuki Hamada

**Affiliations:** 1 Department of Chemistry, Graduate School of Science and Engineering, Kagoshima University, 1-21-35 Korimoto, Kagoshima, 890-0065 Japan; 2 Faculty of Science, Kagoshima University, 1-21-35 Korimoto, Kagoshima, 890-0065 Japan; 3 Department of Translational Research, Joint Research Center for Human Retrovirus Infection, Kagoshima University, 8-35-1 Sakuragaoka, Kagoshima, 890-8544 Japan; 4 Department of Chemistry, Biotechnology, and Chemical Engineering, Graduate School of Science and Engineering, Kagoshima University, 1-21-40 Korimoto, Kagoshima, 890-0065 Japan; 5 Institute for Materials Chemistry and Engineering, Kyushu University, 744 Motooka Nishi-ku, Fukuoka, 819-0395 Japan; 6 Drug Discovery Structural Biology Platform Unit, Center for Biosystems Dynamics Research, RIKEN, 1-7-22 Suehiro, Tsurumi, Yokohama, Kanagawa 230-0045 Japan; 7 Division of Infection Control Research, Center for Advanced Science Research and Promotion, Kagoshima University, 1-21-24, Korimoto, Kagoshima 890-8580 Japan

**Keywords:** *Ocimum tenuiflorum* L., SARS-CoV-2 main protease, M^pro^ inhibitor, Sulfoquinovosyl diacylglycerol

## Abstract

The persistence of the novel severe acute respiratory syndrome coronavirus 2 (SARS-CoV-2) and the emergence of new mutant strains continue to present a substantial threat with potential for future pandemics. Safe, effective, and readily available COVID-19 therapeutics are urgently needed to prepare for future coronavirus pandemics. To help identify new antiviral agents, the present study focused on natural products in the extracts of Holy Basil, *Ocimum tenuiflorum* L., which show potential inhibitory effects against the SARS-CoV-2 main protease (M^pro^). Bioassay-guided isolation of the MeOH extracts of *O. tenuiflorum* led to the identification of a sulfur-containing glyceroglycolipid, sulfoquinovosyl diacylglycerol (SQDG: **1**), as a potent M^pro^ inhibitor that effectively inhibited M^pro^ activity (IC_50_: 0.42 µM). SQDG (**1**) also markedly suppressed SARS-CoV-2 replication (EC_50_, 51.2 µM) *in vitro* while displaying no cytotoxicity (CC_50_ > 100 µM). Further inhibition kinetic studies and docking simulations clearly demonstrated that SQDG strongly inhibited SARS-CoV-2 M^pro^ *in* a competitive and mixed-inhibition manner. These findings highlight SQDG as a promising lead compound for COVID-19 therapy and emphasize the need to explore new drugs from natural sources.

**Graphical Abstract:**

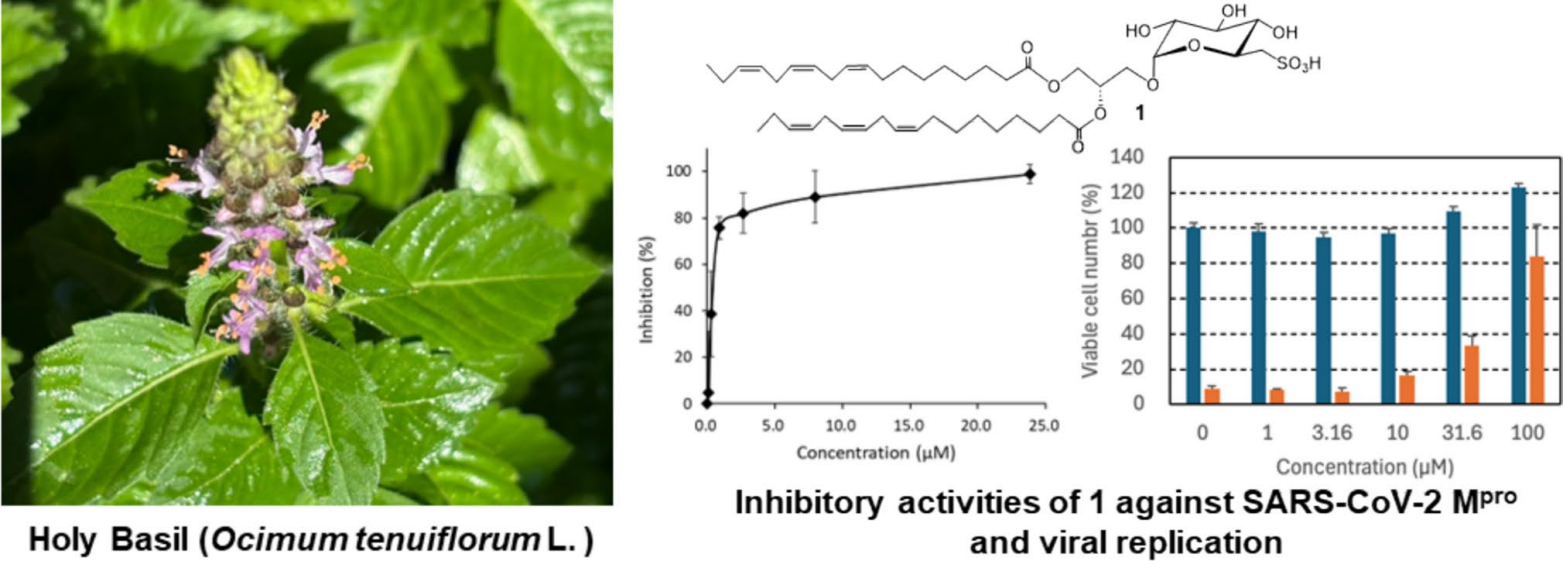

**Supplementary Information:**

The online version contains supplementary material available at 10.1007/s11418-024-01855-6.

## Introduction

The coronavirus disease 2019 (COVID-19) pandemic, caused by the novel severe acute respiratory syndrome coronavirus 2 (SARS-CoV-2), which emerged in December 2019, has significantly impacted global health [1]. World Health Organization (WHO) declared COVID-19 a public health emergency in January 2020 and later, in May 2023, declared the end of the emergency phase. However, owing to the persistence of the virus and the emergence of new mutant strains [2–4], COVID-19 continues to pose a significant threat with potential for future pandemics. Therefore, the development of safer and more effective therapies for COVID-19 remains an urgent priority.

The development of new anti-coronaviral drugs focuses on targeting two key enzymes: RNA-dependent RNA polymerase and the main protease (M^pro^ or 3-chymotrypsin-like protease; 3CL^pro^) [5, 6]. M^pro^ plays a crucial role in viral replication by cleaving polypeptides translated from the RNA of SARS-CoV-2 [7]. M^pro^ shows glutamine-specific cleavage activity not present in human proteases [8, 9] and is highly conserved across coronaviruses, such as SARS and Middle East respiratory syndrome [10], making it an ideal target for drug discovery [11]. M^pro^-specific inhibitors are predicted to have negligible off-target activity and thus have limited side effects [12–14]. These features make M^pro^ an attractive therapeutic target for COVID-19 treatment. Two M^pro^ inhibitors, nirmatrelvir [11] and ensitrelvir [15], have recently been developed. However, nirmatrelvir requires the concurrent use of ritonavir as a metabolic modifier, whereas ensitrelvir still has side effects. Therefore, there is an urgent need to develop more effective and specific anti-SARS-CoV-2 drugs.

Although synthetic M^pro^ inhibitors represent significant advances in COVID-19 therapy, the exploration of natural compounds provides a complementary approach. Leveraging biodiversity to discover novel antiviral agents is crucial for the development of effective treatments. Natural compounds from medicinal plants remain one of the major sources for the discovery of lead compounds for drug development. Recently, some natural compounds originating from medicinal plants have been found to exhibit M^pro^ inhibitory activity [16, 17], encouraging us to identify more potent SARS-CoV-2 M^pro^ inhibitors from herbal medicines. In this context, Holy Basil (*Ocimum tenuiflorum* L., syn. *O. sanctum*) or Tulsi, known for its extensive medicinal properties, emerges as a promising candidate. Holy Basil, cultivated in Southeast Asia for religious and medicinal purposes [18], has various properties, including antifertility, anticancer, antidiabetic, antifungal, antimicrobial, cardioprotective, analgesic, antispasmodic, and adaptogenic effects [19]. Its extracts are used to treat bronchitis, malaria, diarrhea, dysentery, skin conditions, arthritis, eye diseases, and insect bites [20]. A recent study also demonstrated the therapeutic potential of Holy Basil against SARS-CoV-2 infection. Specifically, eugenol, a phenolic compound found in this basil, inhibited the interaction between SARS-CoV-2 spike S1 and ACE2 to induce therapeutic responses [21].

**Fig. 1:**
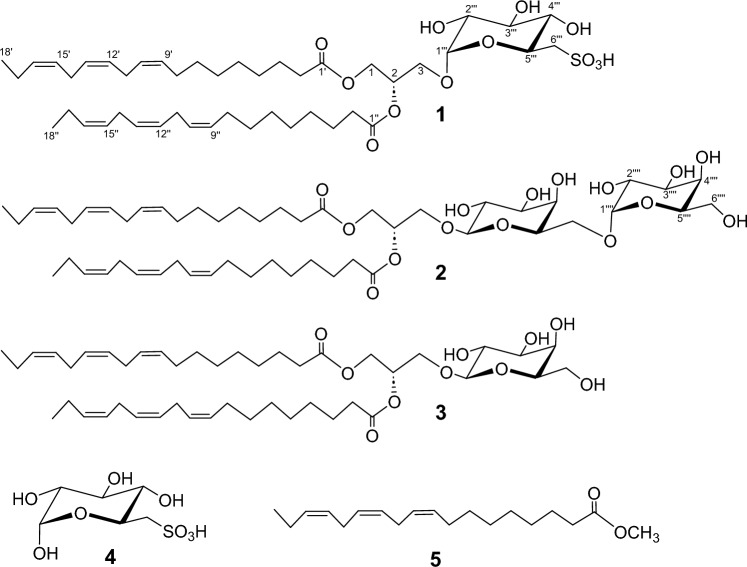
Structures of 1–5

In this study, we aimed to isolate and purify the active components of the methanol extract of the dried aerial parts of *O. tenuiflorum* using a bioassay-guided method and test its efficacy against SARS-CoV-2 M^pro^. We identified a sulfur-containing glyceroglycolipid, sulfoquinovosyl diacylglycerol (SQDG: **1**) (Fig. 1), which inhibits SARS-CoV-2 M^pro^ activity. SQDG (**1**) also inhibited the replication of SARS-CoV-2 *in vitro*. We also performed kinetic analysis and docking simulation to examine the inhibitory mechanisms of SQDG (**1**) against SARS-CoV-2 M^pro^, with the goal of aiding the development of new SARS-CoV-2 inhibitors.

## Results and discussion

### Bioassay-guided purification from *O. tenuiflorum*

**Fig. 2:**
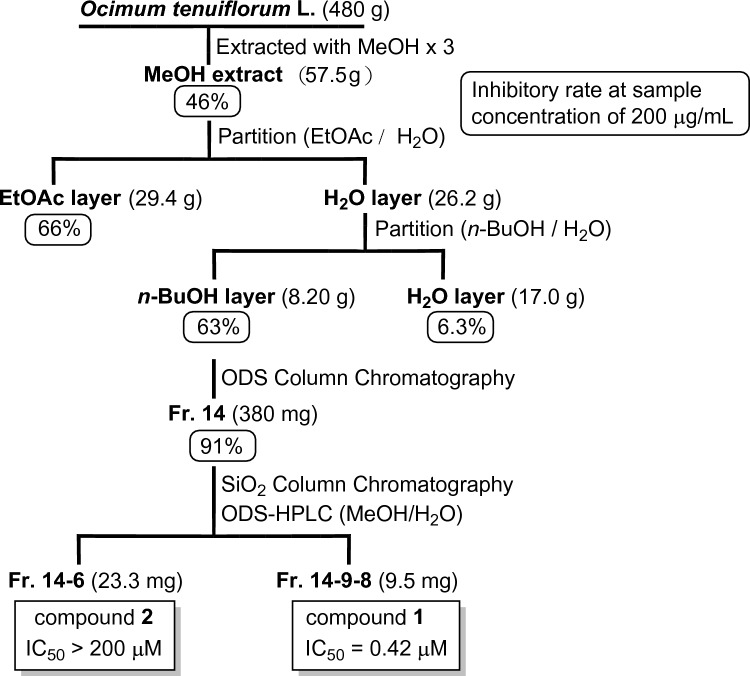
Bioassay-guided isolation scheme from dried aerial parts of Holy Basil, *Ocimum tenuiflorum* L. Inhibitory rate (%) against SARS-CoV-2 M^pro^ is indicated below each of the respective extracts, fractions, and compounds

Upon screening the natural products for inhibitory effects against SARS-CoV-2 main protease M^pro^, the crude methanol extract of the aerial parts of *O. tenuiflorum* showed 46% inhibition at a sample concentration of 200 µg/mL (Fig. 2). The methanol extract was partitioned between H_2_O and EtOAc and the H_2_O-soluble portion was further partitioned between *n*-BuOH and H_2_O. The *n*-BuOH extract, showing 63% inhibition at 200 μg/mL, was subjected to ODS vacuum column chromatography, further yielding 16 fractions. Among these, the 14th fraction exhibited ~91% inhibition at 200 µg/mL. Further fractionation and purification of the 14th fraction using a SiO_2_ column and reverse-phase HPLC yielded compound **1** (fr. 14-9-8, 9.5 mg) and **2** (fr. 14-6, 23.3 mg). Although the chemical structures of the two isolated compounds (**1** and **2**) belonged to the glycolipid family and appeared to be very similar, their M^pro^ inhibitory activities showed extremely different outcomes; the IC_50_ values were 0.42 and >200 µM, respectively. To understand these findings, we solved the chemical structures of **1** and **2** and compared the M^pro^ inhibitory activities of these compounds and related analogs.

### Structures of 1 and 2

**Fig. 3:**
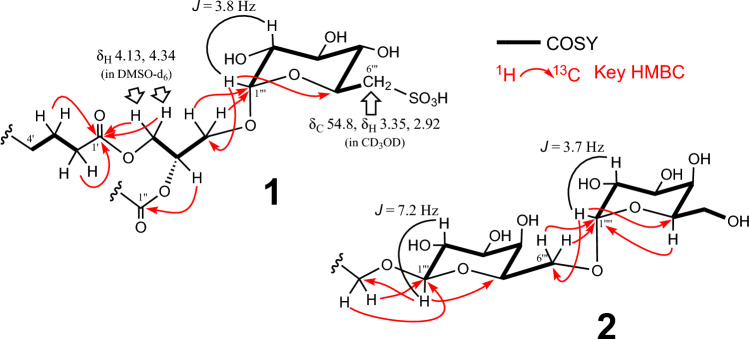
Partial structures leading to the structures of compounds **1** and **2** generated from a combination of ^3^*J* value, ^1^H-^1^H COSY, and HMBC spectral data

Compound **1** was isolated as a colorless, amorphous solid. High-resolution fast atom bombardment mass spectrometry (HR-FAB-MS) analysis of compound **1** in positive-ion mode displayed an [M + H]^+^ ion peak at *m/z* 839.4983, along with an [M+ Na]^+^ ion peak at *m/z* 861.6 (Online Resource Fig. S1), indicating a molecular formula of C_45_H_74_O_12_S. ^1^H-NMR and attached proton test (APT) spectra of **1** (Online Resource Figs. S2 and S3) showed signals corresponding to glycerol, carbohydrate, and unsaturated fatty acid components. Extensive NMR analysis (Fig. 3) was conducted using COSY (Online Resource Fig. S6), HMQC (Online Resource Fig. S4), and HMBC (Online Resource Fig. S5). Based on 2D NMR spectra, C-1 (δ_C_ 64.8 / δ_H_ 4.17 and 4.49), C-2 (δ_C_ 72.2 / δ_H_ 5.28), and C-3 (δ_C_ 67.7/δ_H_ 3.56 and 4.06) were assigned to the glycerol moiety. The other signals at δ_H_ 2.9–4.8 and δ_C_ 54–101 were assigned to one glycosyl residue. The ^1^H-^1^H COSY correlations revealed the presence of one consecutive spin system, H-1‴ to H-6‴, with a quinovosyl unit. The α-configuration of anomeric carbon (δ_C_ 100.6; C-1‴) was determined from the three bond coupling constant values (3.8 Hz) between H-1‴ (δ_H_ 4.75) and H-2‴ (δ_H_ 3.40). The other configurations of the quinovosyl unit were determined from the coupling constant of each proton. HSQC (Online Resource Fig. S4) confirmed the presence of the CH_2_-S group in the sulfodeoxyhexosyl unit, with CH_2_-6″ resonances at an unusually high frequency at δ_C_ 54.8 / δ_H_ 2.92 and 3.35. The HMBC correlations (Online Resource, Fig. S5) of H-1‴ with C-3 and H_2_-3 with C-1‴ confirmed that C-3 was O-glycosylated by sulfoquinovosyl groups. Furthermore, the HMBC correlations of H_2_-1 with C-1′ and H-2 with C-1″ indicated that CH_2_-1 and CH-2 were esterified by the fatty acids. The presence of lipids was confirmed by ^13^C signals at δ_C_ 15.2 (CH_3_-18′) and from δ_C_ 22.0 to 35.5 (CH_2_) with several double bond signals at δ_C_ 128–133/δ_H_ 5.27–5.39. Based on this structural information, compound **1** was identified as a SQDG. The presence of α-linolenic acid (C_18:3_) was confirmed using GC-MS. The stereochemistry of C-2*S* of glycerol and D-sulfoquinovose was determined by comparing the optical rotation value ([α]_D_ 48.2°) and the chemical shifts of glycerol H-1 (δ_H_ 4.13 and 4.34) in DMSO-*d*_6_ (Online Resource Fig. S8) with those reported previously [22]. This comparison confirmed that the chemical structure of compound **1** was 1,2*S*-di-(9*Z*,12*Z*,15*Z*-octadecatrienoyl)-3-(6′-sulfo-α-d-quinovosyl)-*sn*-glycerol.

Compound **2** was obtained as a colorless amorphous solid. FAB-MS analysis in the positive-ion mode of compound **2** displayed [M + H]^+^ and [M + Na]^+^ ion peaks at *m/z* 937.6 and 959.6, respectively (Online Resource Fig. S9), which corresponds to the molecular formula C_51_H_84_O_15_. The ^1^H-NMR and APT signals for compound **2** (Online Resource Figs. S10 and S11) were similar to those of compound **1**, except for the carbohydrate region. The HSQC (Online Resource Fig. S12) revealed two anomeric signals at δ_C_ 105.8/δ_H_ 4.24 (*J* = 7.2 Hz; H-1‴) and δ_C_ 101.2/δ_H_ 4.86 (*J* = 3.7 Hz; H-1‴′), along with two CH_2_ signals at δ_C_ 68.3/δ_H_ 3.66 and 3.91 and at δ_C_ 63.4/δ_H_ 3.69, corresponding to CH_2_-6‴ and CH_2_-6‴′, respectively (Fig. 3). These findings suggested the presence of an α-galactosyl-β-(1→6)-galactosyl unit. These results confirmed that compound **2** was a digalactosyl diacylglycerol (DGDG). GC-MS analysis of compound **2** after hydrolysis-methyl esterification revealed the structure of the fatty acids to be α-linolenic acid (C_18:3_). DGDG and monogalactosyl diacylglycerol (MGDG; **3**) along with SQDG (**1**) are significant metabolites commonly found in the chloroplast thylakoid membranes of photosynthetic plants (approximately 90% of the total lipid molecules in the thylakoid membrane) [23]. Assuming that the absolute stereochemistry of the sugar and glycerol parts in **2** were the same, the chemical structure of compound **2** was determined to be 1,2-di-(9*Z*,12*Z*,15*Z*-octadecatrienoyl)-3-*O*-(α-d-galactosyl-1-6-β-d-galactosyl)-*sn*-glycerol.

### Inhibitory activity on M^pro^

**Fig. 4:**
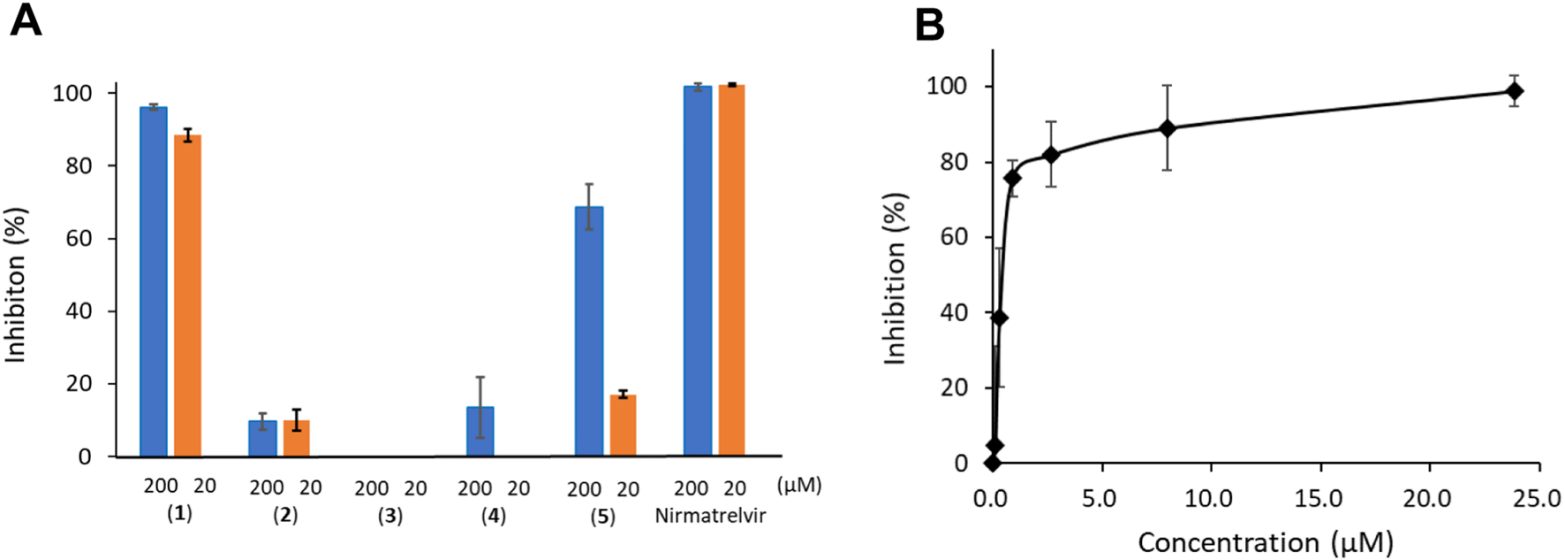
Inhibitory activities of SQDG (**1**), DGDG (**2**), MGDG (**3**), sulfoquinovose (**4**), and methyl linolenate (**5**) against the SARS-CoV-2 main protease. **A** Compounds were evaluated at two concentrations (20 and 200 μΜ). **B** SQDG was serially diluted and its SARS-CoV-2 main protease inhibitory activity was assessed. All experiments were conducted in triplicate and the mean values ± standard deviations are shown

We evaluated the inhibitory activities of SQDG (**1**) and DGDG (**2**) against SARS-CoV-2 M^pro^, along with those of the commercially available compounds MGDG (**3**), sulfoquinovose (**4**), and methyl linolenate (**5**) (Fig. 4A). Enzymatic assays were performed at concentrations of 20 and 200 µM for all compounds. Among compounds **1**–**3**, which belong to the glyceroglycolipid family, only compound **1** demonstrated significant potency, with an M^pro^ inhibition percentage of 88.5% and an IC_50_ value of 0.42 ± 0.15  µM (Fig. 4B). DGDG (**2**) and MGDG (**3**) had virtually no effect, even at 200 µM, indicating that the sulfoquinovose moiety of SQDG is essential for M^pro^ inhibition. Sulfoquinovose (**4**) and methyl linolenate (**5**) displayed very weak M^pro^ inhibitory activity, although the IC_50_ value of **5** was approximately 100 µM. These results indicated that the combined structure of the sulfate group and fatty acids in SQDG plays a crucial role in its potent M^pro^ inhibitory activity.

### Kinetic analysis of SQDG on M^pro^

**Fig. 5:**
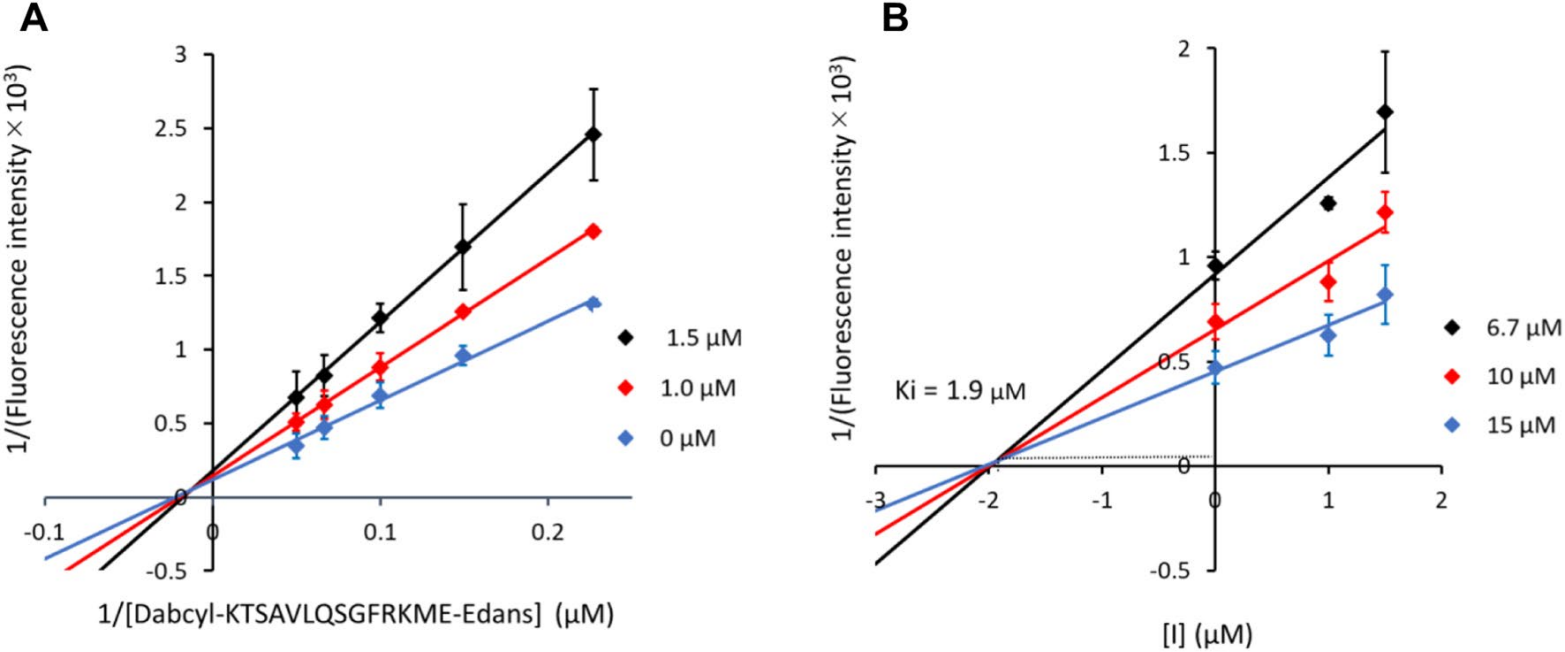
Lineweaver-Burk plot (**A**) and the Dixon plot (**B**) of SQDG (**1**) against SARS-CoV-2 M^pro^. The SQDG concentrations of 0, 1.0, and 1.5 µM were used in the Lineweaver–Burk plot (**A**), and the substrate concentrations of 6.7, 10, and 15 µM were used in the Dixon plot (**B**). All experiments were conducted in triplicate and the mean values ± standard deviations are shown

**Fig. 6:**
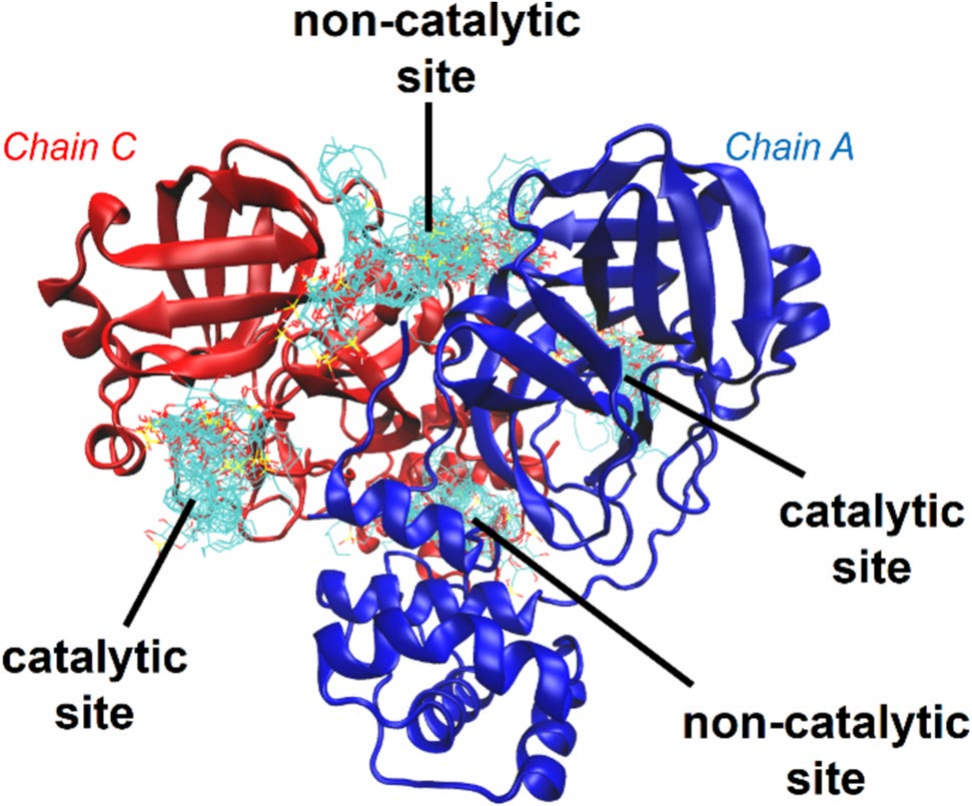
Predicted binding structures of SQDG (**1**) using AutoDock Vina. A total of 100 predicted binding structures across the entire M^pro^ dimer were obtained. Chains A and B are shown as blue and red ribbon representations, respectively, and SQDG (**1**) is shown as a colored stick representation

SQDG exhibited strong M^pro^ inhibitory activity; however, its mode of inhibition differed from that of common M^pro^ inhibitors, such as nirmatrelvir and ensitrelvir. This discrepancy may be attributed to the structural differences between these inhibitors and SQDG. To further investigate the inhibitory mechanisms of SQDG (**1**), an inhibition kinetics study was conducted. As shown in Fig. 5, the Lineweaver–Burk plot of **1** against SARS-CoV-2 M^pro^ demonstrated that compound **1** strongly inhibited M^pro^ in a mixed-inhibition manner. The Dixon plot indicates a *k*_*i*_ value of 1.9 μM with a competitive mode of inhibition. These results suggest that SQDG (**1**) acts as a mixed-type inhibitor of SARS-CoV-2 M^pro^, implying that **1** may bind to multiple locations on this key target enzyme. These results were also supported by docking simulations of compound **1** on a dimer crystal structure of SARS-CoV-2 M^pro^ [24], i.e., four pockets (two catalytic sites of both chains and two non-catalytic sites located at the dimer interface) were identified as possible ligand-binding pockets of this key enzyme (Fig. 6 and Online Resource Fig. S16). These results indicate that the mode of inhibition, as shown in the Lineweaver plot, is a mixed-type.

### Inhibitory effect on viral replication *in vitro*

**Fig. 7:**
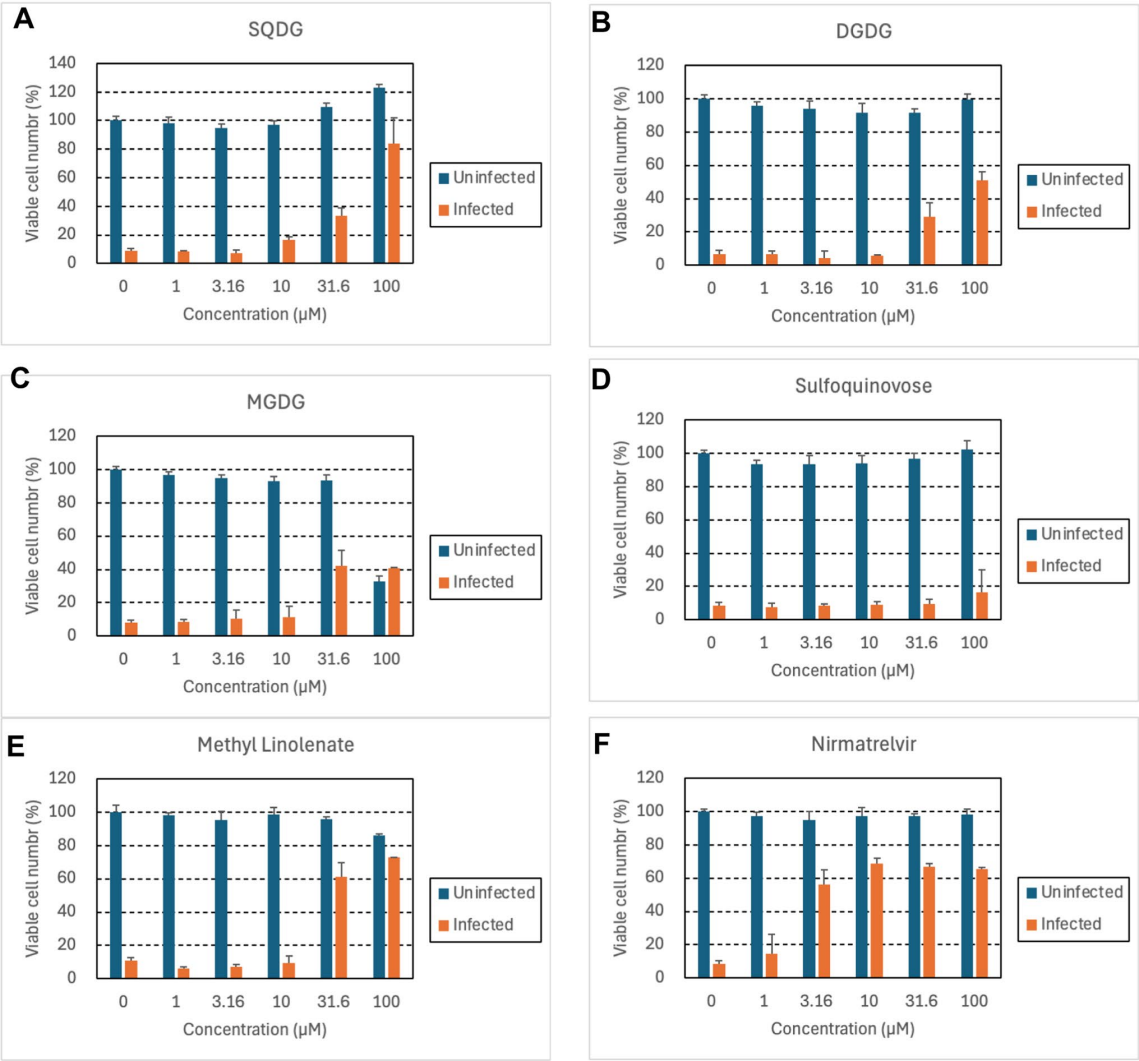
Inhibitory effect of compounds on SARS-CoV-2 replication in VeroE6/TMPRSS2 cells. The cells were mock-infected (blue columns) or infected with SARS-CoV-2 at an MOI of 0.002 (red columns) and cultured in the presence of the indicated concentrations of SQDG (**A**), DGDG (**B**), MGDG (**C**), sulfoquinovose (**D**), methyl linolenate (**E**), or nirmatrelvir (**F**). After three days, cell viability was assessed using the tetrazolium dye method. All experiments were conducted in triplicate and the mean values ± standard deviations are shown

To further explore the therapeutic potential of SQDG against SARS-CoV-2 infection, we assessed the inhibitory effects of compounds **1–5** on viral replication (Fig. 7). When VeroE6/TMPRSS2 cells were infected with SARS-CoV-2 and left untreated for 3 days, the virus-induced cytopathic effect caused near-complete destruction of the cells. SQDG (**1**) showed dose-dependent protection of the infected cells from virus-induced cell destruction, with an EC_50_ value of 51 µM (Fig. 7A), while this compound did not show apparent cytotoxicity at concentrations up to 100 µM. These results indicated that SQDG selectively inhibits SARS-CoV-2 replication. It is important to note that amphiphilic compounds such as SQDG may not be fully internalized into cells, potentially leading to lower antiviral activity compared to their inhibitory activity against the M^pro^ enzyme. Compounds **2** and **5** also inhibited cell death caused by SARS-CoV-2 in a concentration-dependent manner (Fig. 7B, E); however, their inhibitory activity against M^pro^ was weak. This suggests that other mechanisms, such as viral adsorption or inhibition of intracellular entry, may be involved in suppressing viral replication.

This study found that SQDG isolated from Holy Basil, *O. tenuiflorum*, displayed strong inhibition of SARS-CoV-2 M^pro^ and demonstrated concentration-dependent inhibition of SARS-CoV-2-induced cell death. Previous studies have shown that SQDGs exhibit strong antiviral against human immunodeficiency virus (HIV), antitumor, and anti-inflammatory activities [25, 26]. For example, SQDG identified in cyanobacteria (blue-green algae) exhibits antiviral activity [27], whereas those obtained from the marine red alga *Gigartina tenella* and pteridophyte *Athyrium niponicum* have been shown to inhibit the activities of DNA polymerase and HIV reverse transcriptase [28, 29]. Similarly, SQDG from spinach (*Spinacia oleracea* L.) inhibited DNA polymerase and suppressed the proliferation of human gastric cancer cells (NUGC-3) [30]. These findings suggest that SQDG may interact with other enzymes, such as DNA- or RNA-polymerase, which could contribute to its ability to inhibit SARS-CoV-2 replication.

In conclusion, the present study demonstrates that SQDG inhibits SARS-CoV-2 M^pro^ with an IC_50_ value of 0.42 µM and effectively suppresses viral replication. While the development of a novel therapeutic agent from SQDG against COVID-19 is complex, discovering a compound that selectively inhibits SARS-CoV-2 replication is highly significant. Further research into the structure-activity relationship and mechanism of action of this sulfosaccharide-containing glyceroglycolipid could pave the way for new therapeutic agents, not only for COVID-19 but also for other infectious diseases.

## Experimental section

### General procedures

Optical rotation was measured at 25°C using a JASCO P-1030 Polarimeter (Jasco International Co. Ltd., Tokyo, Japan). NMR spectra were acquired at 300 K using a JEOL JNM-ECA 600 (JEOL Ltd., Tokyo, Japan) and Avance III 600 Cryo-probe Spectrometers (Bruker BioSpin Group, Faellanden, Switzerland). UV–VIS and IR spectra were recorded on Shimadzu UV-2700 (Shimadzu Co., Kyoto, Japan) and JASCO IRT-3000 Spectrometer equipped with an ATR-30-Z accessory (Jasco International Co. Ltd., Tokyo, Japan), respectively. FAB mass spectra were obtained using a JEOL JMS-700 MStation system (JEOL Ltd.). Column chromatography was performed using silica gel 60 (Merck, 70–230 mesh) and Cosmosil 75C_18_-OPN (Nacalai Tesque, Kyoto, Japan), and thin-layer chromatography was performed using silica gel 60 F254 plates (Merck, 0.25 mm thick). HPLC was performed using a Shimadzu LC-10AT instrument equipped with an SPD-20A detector. The HPLC separation was performed using a C18 column, Cosmosil 5C_18_-MS-II (Nacalai Tesque; 10 mmI.D. × 250 mm) (Fig. 1).

### Materials

Holy basil (*Ocimum tenuiflorum* L.) was collected from farms managed by Botanical Factory Co. Ltd. (www.botanical.co.jp, Japan), Minami-Osumi-cho, Kagoshima Prefecture, Japan, in the early summer of 2023. Fresh samples were dried naturally and frozen at −30°C until extraction. Compound **4**, Sulfoquinovose (CAS No.: MC9551), was purchased from MCAT GmbH (Donaueschingen, Germany). Compound **3** (MGDG; CAS No.: 1932659-76-1) and Compound **5** (Methyl linolenate; CAS No.: 301-00-8) were purchased from Sigma-Aldrich (Tokyo, Japan). Supelco 37 Component FAME Mix (CAS No.: CRM47885) was used as the methylated fatty acid standard.

### Extraction and isolation

The dried plants (480 g) were homogenized with MeOH (3 L × 3), and the extract was concentrated under reduced pressure at 40–45°C. The residue (57.5 g) was partitioned between EtOAc (3 L) and H_2_O (1 L), and the H_2_O layer (26.2 g) was further partitioned between *n*-BuOH (3 L) and H_2_O. The *n*-BuOH layer (8.20 g), which showed M^pro^ inhibitory activity, was subjected to vacuum column chromatography (ODS, mobile phase: stepwise elution with 50% MeOH/H_2_O –80% MeOH/H_2_O –100% MeOH–10% CH_2_Cl_2_/MeOH–100% CH_2_Cl_2_) to obtain 16 fractions. Among these 16 fractions, Fraction 14 (fr. 14; 380 mg) exhibited the most potent inhibitory activity. Therefore, further fractionation of fr. 14 was performed using flash column chromatography (SiO_2_, mobile phase: 20% EtOAc /*n*-hexane –100% EtOAc) followed by reverse-phase HPLC, resulting in the isolation of compound **2** (fr. 14-6; 23.3 mg) and compound **1** (fr. 14-9-8; 9.5 mg).

### Measurement of fatty acids using GC-MS

Methyl esterification of the fatty acids in **1** and **2** (each 1.0 mg) and purification of the FAMEs were conducted using the Nacalai Tesque fatty acid methylation kit (p/N: 06482-04) and a methylated fatty acid purification kit (P/N: 06483-94) following the manufacturers’ instructions. The resultant solutions were allowed to evaporate under a stream of N_2_ gas and measured using GC-MS after dissolution in 1 mL of *n*-hexane. GC-MS analysis of the FAMEs was performed on a 7890A/5975C GC/MSD system (Agilent Technologies, CA, USA) equipped with a DB-WAX capillary column (J&W Scientific Inc., CA, USA; 30 m × 0.25 mm I.D., 0.25 µm film thickness). The injection port was then heated to 250°C. The column was heated to 50°C for 5 min, and the temperature was increased at 10°C/min to a final temperature of 240°C, which was maintained for 10 min. The MS conditions were as follows: ionization voltage, 70 eV; emission current, 40 mA; mass range, 50–800 amu; scan rate, 1.0 scan/s; split ratio, 5:1. Helium was used as the carrier gas at a flow rate of 1 mL/min. FAMEs were identified by comparing the retention times of FAMEs with those of a standard mixture and by matching the mass spectra with the NIST Mass Spectral Library.

#### 1,2-di-(9*Z*,12*Z*,15*Z*-octadecatrienoyl)-3-(6′-sulfo-α-d-quinovosyl)-*sn*-glycerol (SQDG) (1)

Colorless amorphous solid; ${[\alpha ]}_{D}^{20}$ +48.2 (*c* 1.67); IR (film): 3432, 1733, 1645, 1374, 1171, 1034 cm^-1^; ^1^H and ^13^C NMR (CD_3_OD) see Table 1; HR-FAB-MS *m/z* 839.4983 [M+H]^+^ (calculated for C_45_H_75_O_12_S, 839.4987).

**Table 1:** NMR spectral data for compounds **1** and **2** in CD_3_OD at 300 K

No.	**1**		**2**	
	δ_C_ (mult.)	δ_H_ (mult., *J *=Hz)	δ_C_ (mult.)	δ_H_ (mult., *J *=Hz)
1	64.8 (t)	4.49 (dd, 12.0, 2.9)	64.5 (t)	4.43 (dd, 12.1, 2.7)
		4.17 (dd, 12.0, 6.8)		4.22 (dd, 12.1, 6.6)
2	72.2 (d)	5.28^a^	72.27 (d)	5.24 (m)
3	67.7 (t)	4.08 (brdd, 10.7, 5.3)	69.3 (t)	3.93 (dd, 10.9, 5.4)
		3.56 (dd, 10.7, 6.2)		3.72^a^
1' or 1"	175.4 or 175.6 (s)	–	175.5 or 175.6 (s)	–
2' or 2"	35.5 (t)	2.34 (t, 7.6) or 2.32 (t, 7.6)	35.5 (t)	2.32 (t, 7.6) or 2.31 (t, 7.6)
3', 3"	26.6 (t)	1.60 (m)	26.6 (t)	1.60 (m)
4', 4"	30.8 (t)	1.32^a^	30.8 (t)	1.32^a^
5', 5"	30.9–31.0^a^ (t)	1.28–1.30^a^	30.9–31.0^a^ (t)	1.28–1.30^a^
6', 6"	31.2 (t)	1.28–1.30^a^	31.2 (t)	1.28–1.30^a^
7', 7"	31.3 (t)	1.34^a^	31.3 (t)	1.34^a^
8', 8"	28.7 (t)	2.08^a^	28.7 (t)	2.06^a^
9', 9"	129.4–131.6^a^ (d)	5.27–5.35^a^	128.8–131.6^a^ (d)	5.26–5.36^a^
10', 10"	129.4–131.6^a^ (d)	5.27–5.35^a^	128.8–131.6^a^ (d)	5.26–5.36^a^
11', 11"	27.1 (t)	2.80 (t, 6.0)	27.1 (t)	2.80 (t, 6.0)
12', 12"	129.4–131.6^a^ (d)	5.27–5.35^a^	128.8–131.6^a^ (d)	5.26–5.36^a^
13', 13"	129.4–131.6^a^ (d)	5.27–5.35^a^	128.8–131.6^a^ (d)	5.26–5.36^a^
14', 14"	27.0 (t)	2.80 (t, 6.0)	27.0 (t)	2.80 (t, 6.0)
15', 15"	128.8 (d)	5.31^a^	128.8–131.6^a^ (d)	5.26–5.36^a^
16', 16"	133.3 (d)	5.34^a^	133.3 (d)	5.26–5.36^a^
17', 17"	22.0 (t)	2.08^a^	22.0 (t)	2.06^a^
18', 18"	15.2 (q)	0.98 (t, 7.5)	15.2 (q)	0.96 (t, 7.5)
1"'	100.6 (d)	4.75 (d, 3.8)	105.8 (d)	4.24 (d, 7.2)
2"'	74.0 (d)	3.40 (dd, 9.5, 3.8)	72.9 (d)	3.50 (dd, 9.7, 7.2)
3"'	75.4 (d)	3.62 (dd, 9.5, 9.3)	75.2 (d)	3.47 (dd, 9.7, 3.2)
4"'	75.5 (d)	3.09 (dd, 9.5, 9.3)	70.6 (d)	3.87^a^
5"'	70.3 (d)	4.06^a^	75.1 (d)	3.74^a^
6"'	54.8 (t)	3.35^a^	68.3 (t)	3.91^a^
		2.92 (dd, 14.3, 9.1)		3.66 (dd, 10.0, 6.0)
1""			101.2 (d)	4.86 (d, 3.7)
2""			70.8 (d)	3.78 (dd, 10.1, 3.7)
3""			72.0(d)	3.72^a^
4""			71.7 (d)	3.87^a^
5""			73.0 (d)	3.84 (brt, 6.6)
6""			63.4 (t)	3.69^a^

^a^Superimposed by other signals

#### 1,2-di-(9*Z*,12*Z*,15*Z*-octadecatrienoyl)-3-*O*-(α-d-galactosyl-1-6-β-d-galactosyl)-*sn*-glycerol (DGDG) (2)

Colorless amorphous solid; ${[\alpha ]}_{D}^{20}$ +25.2 (*c* 0.15); IR (film): 3393, 1733, 1150, 1070 cm^-1^; ^1^H and ^13^C NMR (CD_3_OD) see Table 1; FAB-MS *m/z* 937.6 [M+H]^+^ and 959.6 [M + Na]^+^.

### ***In vitro*** M^pro^ assays

SARS-CoV-2 M^pro^ was purchased from Sigma-Aldrich (Cat# SAE0172). The fluorogenic substrate (Dabcyl-KTSAVLQ ↓SGFRKME-Edans-NH_2_) for M^pro^ was purchased from the Peptide Institute, Inc., Japan. The inhibition assay relies on fluorescence resonance energy transfer (FRET), employing a fluorescent protein-based substrate established in a previous study [31]. Briefly, a 20 nM portion of purified SARS-CoV-2 M^pro^ was preincubated in a 96-well black plate (Cat#237105; Thermo Scientific, USA) with compounds in 20 mM 4-(2-hydroxyethyl) piperazine-1-ethanesulfonic acid (HEPES) (pH 6.5), 120 mM NaCl, 0.4 mM EDTA, and 4 mM DTT for approximately 5 min before the reaction was initiated by the addition of 10 µM substrate. Protease activity was monitored at 30°C by FRET with excitation and emission wavelengths of 340 and 485 nm, respectively, using a multiplate reader (TECAN Infinite 200, USA). The reduction in fluorescence at 485 nm was analyzed using single exponential decay to obtain the observed rate constant (k_obs_). The relative activity of M^pro^ was calculated as the ratio of k_obs_ to inhibitors compared with that without inhibitors. The relative IC_50_ values of the compounds were determined by fitting the relative activities of different inhibitor concentrations to a four-parameter logistic equation.

### Docking calculations

The crystal structure of M^pro^ (PDB ID: 6XHU [32]) used in this study was obtained from the RCSB PDB. The 2D structure of SQDG (**1**) was downloaded from PubChem (PubChem ID: 15719992, https://pubchem.ncbi.nlm.nih.gov/) in the SDF format, and Open Babel [33] was used to generate the 3D structure. This structure was further optimized by quantum chemical calculations at the B3LYP/6-31G level using the Gaussian 16 software [34]. AutoDock Vina [24] was used as docking software. To search for binding structures of SQDG (**1**) across the entire M^pro^ dimer, five independent docking calculations were performed with different cubic spaces (Fig. S16) as the target region. Each docking calculation was configured to output 100 predicted binding structures (NUM_MODES=100), resulting in a total of 500 predicted binding structures. The top 20 structures obtained from each docking calculation (i.e., a total of 100 structures) are shown in Fig. 6. Four sites within the M^pro^ dimer (two catalytic sites on chains A and B and two non-catalytic sites located at the dimer interface) were identified as potential SQDG (**1**) binding sites.

### Antiviral assays

VeroE6 cell line expressing transmembrane protease serine 2 (VeroE6/TMPRSS2), which is highly susceptible to SARS-CoV-2 infection [35], was used for anti-SARS-CoV-2 assays. Briefly, VeroE6/TMPRSS2 cells (2 × 10^4^ cells/well) were cultured in 96-well microtiter plates and incubated at 37 °C. After 24 h, the cells were mock-infected or infected with SARS-CoV-2 (WK-521 strain, GISAID database ID EPI_ISL_408667) at a multiplicity of infection of 0.002 and cultured in the absence or presence of various concentrations of test compounds. After three days, cell viability was assessed using the tetrazolium dye method [36]. All experiments involving SARS-CoV-2 were conducted at the biosafety level 3 (BSL3) facilities at Kagoshima University.

## Supplementary Information

Below is the link to the electronic supplementary material.

Supplementary file1 (DOCX 3493 KB)
