# Supplementary material for: Sulfoquinovosyl diacylglycerol, a component of Holy Basil Ocimum tenuiflorum, inhibits the activity of the SARS-CoV-2 main protease and viral replication in vitro
Source: J Nat Med. 2024 Nov 25;79(1):122–33. doi: 10.1007/s11418-024-01855-6 (PMC11735596; doi:10.1007/s11418-024-01855-6)
Supplement: Supplementary file 1 — Supplementary file1 (DOCX 3493 KB) [file 11418_2024_1855_MOESM1_ESM.docx]

**Supporting Information**

**Sulfoquinovosyl diacylglycerol, a Component of Holy Basil *Ocimum tenuiflorum*, inhibits the Activity of the SARS-CoV-2 Main Protease and Viral Replication *in vitro***

**Hinako Koze^1,2^, Masayuki Sudoh^3^, Satoaki Onitsuka^1,2^, Hiroaki Okamura^1,2^, Takeshi Ishikawa^4^, Fumito Tani^5^, Yukako Miyata-Yabuki^6^, Mikako Shirouzu^6^, Masanori Baba^7^, Mika Okamoto^7^ and Toshiyuki Hamada^1,2,^***

^1^ Department of Chemistry, Graduate School of Science and Engineering, Kagoshima University, 1-21-35 Korimoto, Kagoshima 890-0065, Japan

^2^ Faculty of Science, Kagoshima University, 1-21-35 Korimoto, Kagoshima 890-0065, Japan

^3^ Department of Translational Research, Joint Research Center for Human Retrovirus Infection, Kagoshima University, 8-35-1 Sakuragaoka, Kagoshima 890-8544, Japan

^4^ Department of Chemistry, Biotechnology, and Chemical Engineering, Graduate School of Science and Engineering, Kagoshima University, 1-21-40 Korimoto, Kagoshima 890-0065, Japan

^5^ Institute for Materials Chemistry and Engineering, Kyushu University, 744 Motooka Nishi-ku, Fukuoka 819-0395, Japan

^6^ Drug Discovery Structural Biology Platform Unit, Center for Biosystems Dynamics Research, RIKEN, 1-7-22 Suehiro, Tsurumi, Yokohama, Kanagawa 230-0045, Japan

^7^ Division of Infection Control Research, Center for Advanced Science Research and Promotion, Kagoshima University, 1-21-24, Korimoto, Kagoshima 890-8580, Japan

**Table of Contents**

**SUPPLEMENTARY FIGURES. 3**

**Figure S1. HR-FAB-MS of 1. 3**

**Figure S2. ^1^H NMR spectrum of 1 in CD_3_OD (600 MHz / 300 K). 3**

**Figure S3. APT spectrum of 1 in CD_3_OD (600 MHz / 300 K). 4**

**Figure S4. HMQC spectrum of 1 in CD_3_OD (600 MHz / 300 K). 4**

**Figure S5. HMBC spectrum of 1 in CD_3_OD (600 MHz / 300 K). 5**

**Figure S6. ^1^H-^1^H COSY spectrum of 1 in CD_3_OD (600 MHz / 300 K). 5**

**Figure S7. IR spectrum of 1. 6**

**Figure S8. ^1^H NMR spectrum of 1 in DMSO-*d*_6_ (600 MHz / 300 K). 6**

**Figure S9. FAB Mass spectrum of 2. 7**

**Figure S10. ^1^H NMR spectrum of 2 in CD_3_OD (600 MHz / 300 K). 7**

**Figure S11. APT spectrum of 2 in CD_3_OD (600 MHz / 300 K). 8**

**Figure S12. HMQC spectrum of 2 in CD_3_OD (600 MHz / 300 K). 8**

**Figure S13. HMBC spectrum of 2 in CD_3_OD (600 MHz / 300 K). 9**

**Figure S14. ^1^H-^1^H COSY spectrum of 2 in CD_3_OD (600 MHz / 300 K). 9**

**Figure S15. IR spectrum of 2. 10**

**Figure S16. Five target regions in M^pro^ dimer for docking calculations of SQDG (1) 10**

**
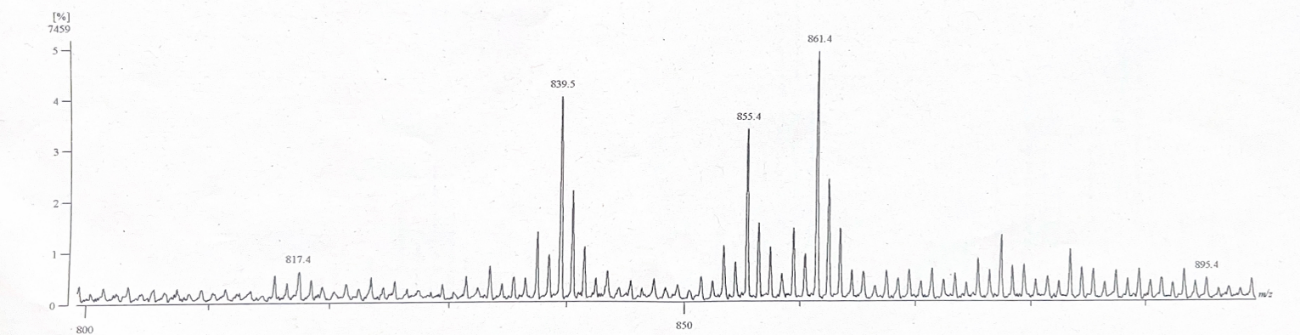
SUPPLEMENTARY FIGURES.**

**
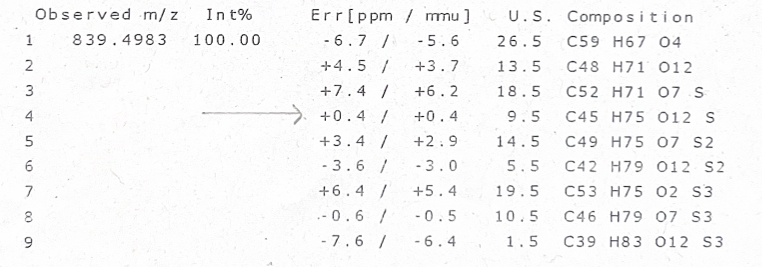
**

**Figure S1. HR-FAB-MS of 1**

**
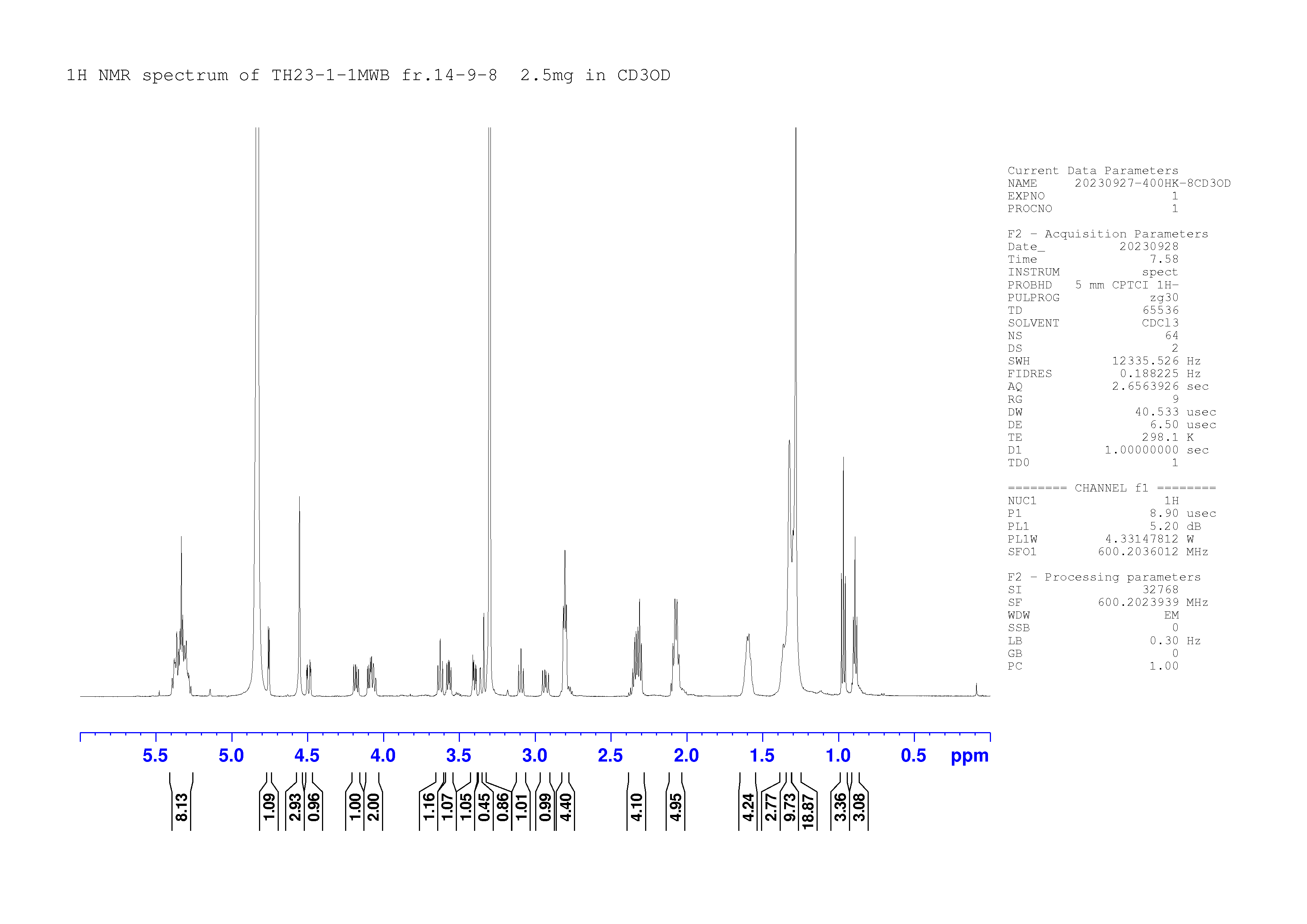
**

**Figure S2. ^1^H NMR spectrum of 1 in CD_3_OD (600 MHz / 300 K)**

**
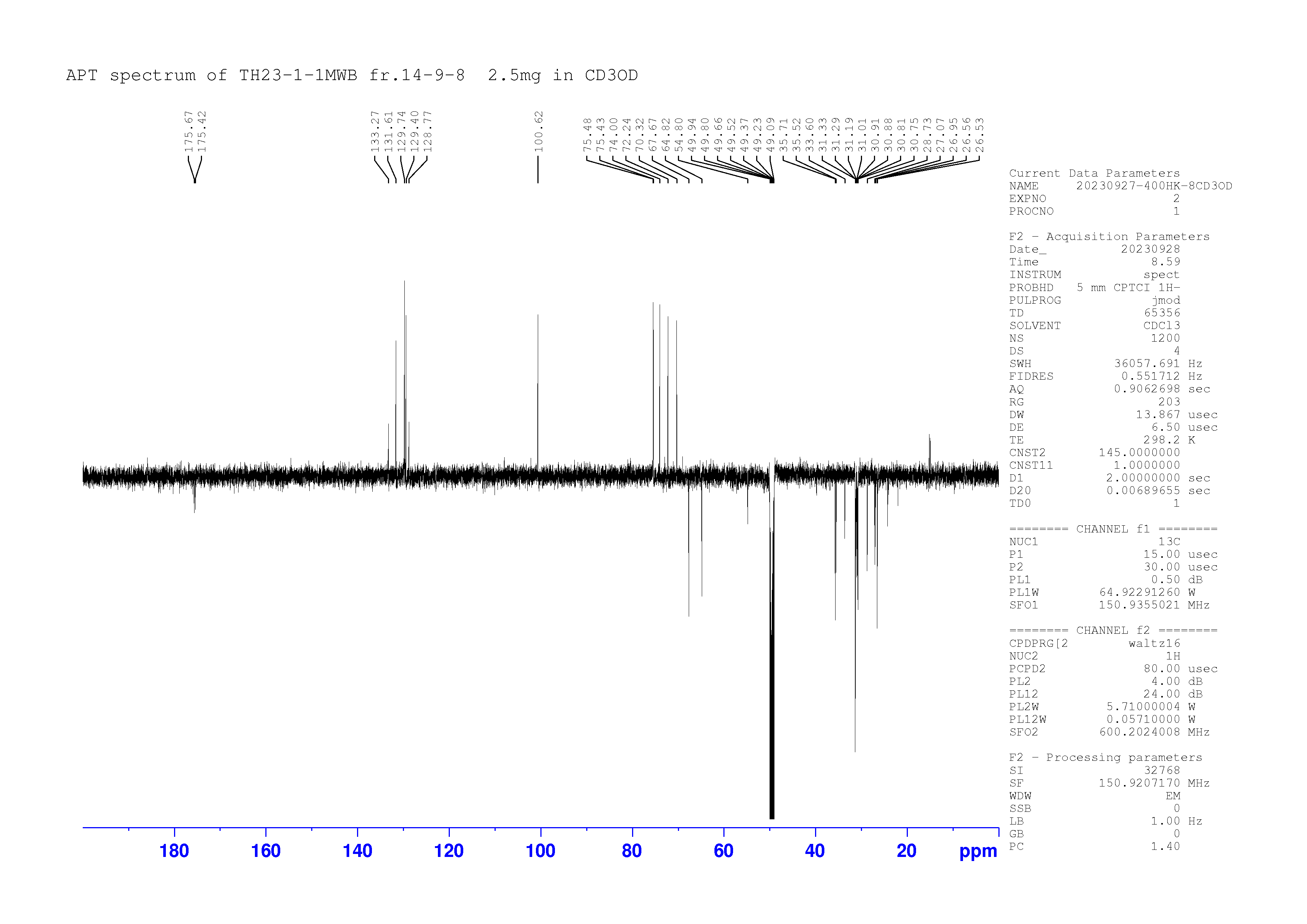
**

**Figure S3. APT spectrum of 1 in CD_3_OD (600 MHz / 300 K)**

**
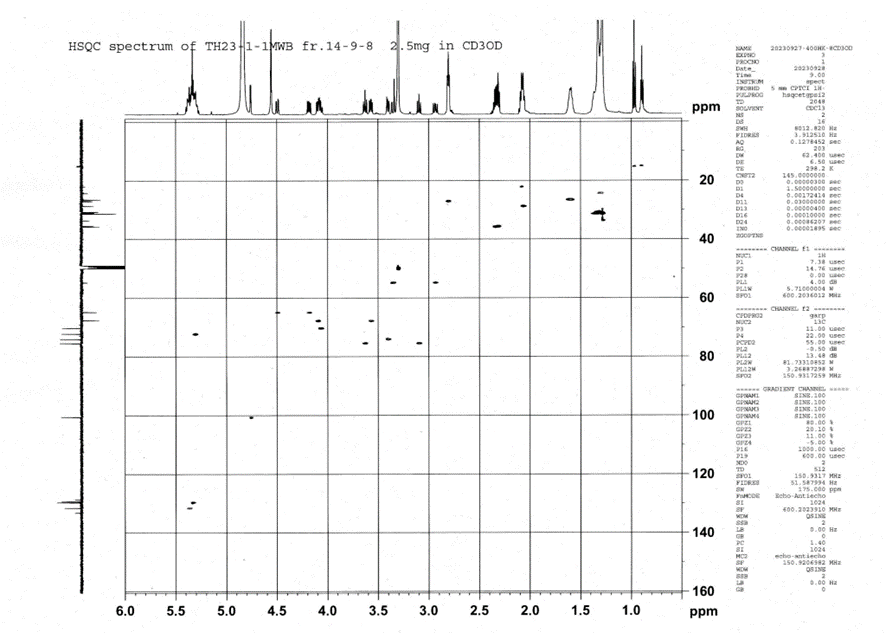
**

**Figure S4. HMQC spectrum of 1 in CD_3_OD (600 MHz / 300 K)**

**
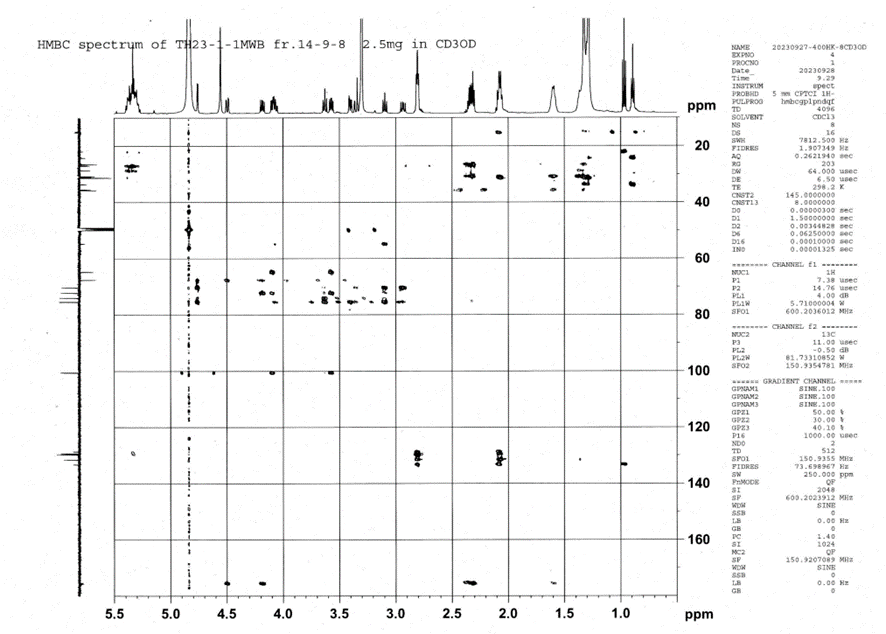
**

**Figure S5. HMBC spectrum of 1 in CD_3_OD (600 MHz / 300 K)**

**
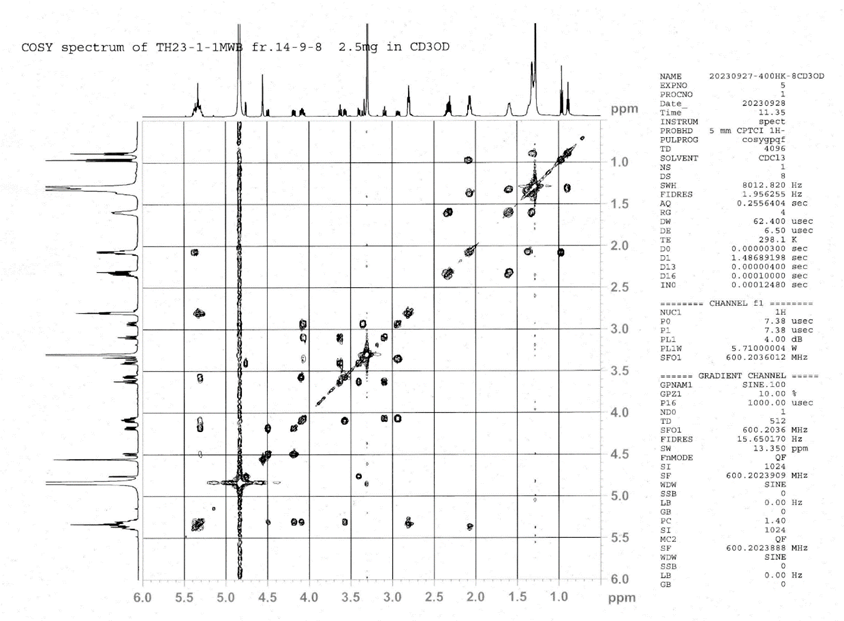
**

**Figure S6. ^1^H-^1^H COSY spectrum of 1 in CD_3_OD (600 MHz / 300 K)**

**
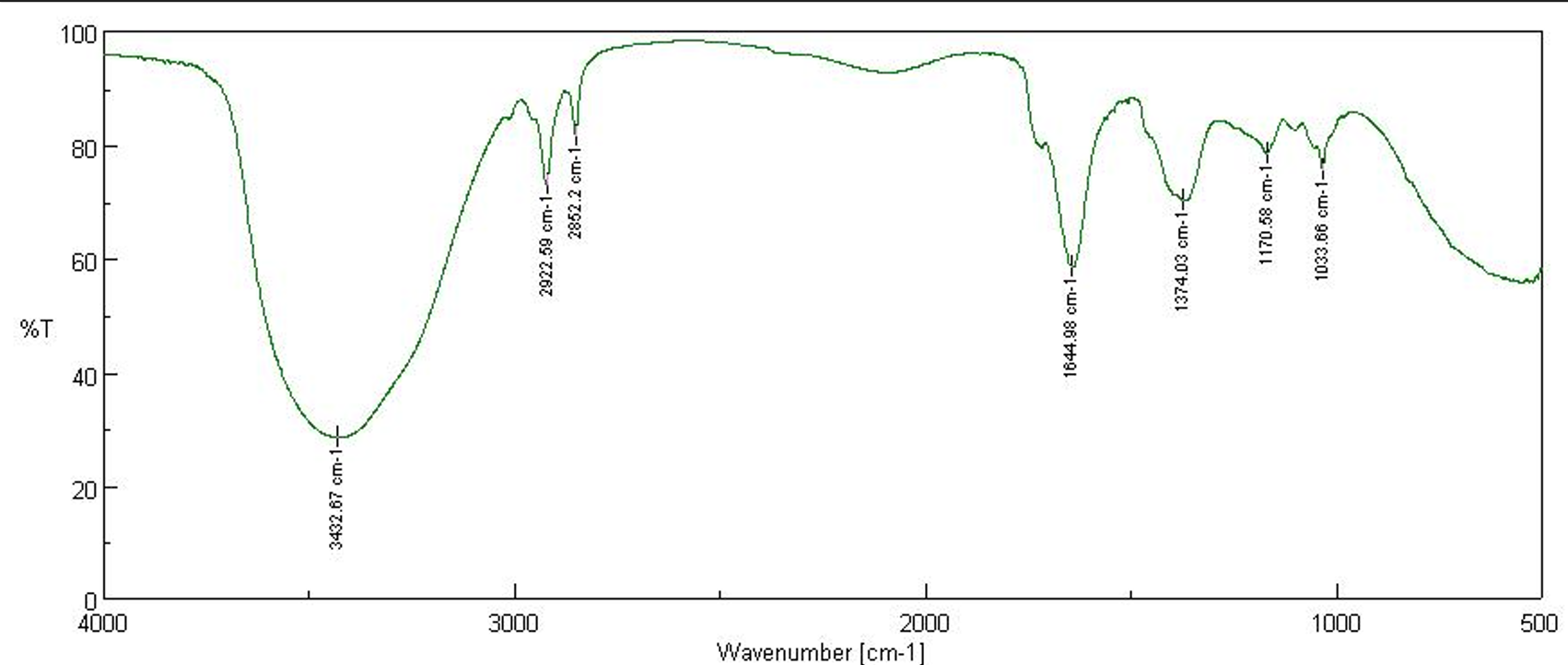
**

**Figure S7. IR spectrum of 1**

**Figure S8. ^1^H NMR spectrum of 1 in DMSO-*d*_6_ (600 MHz)**

**
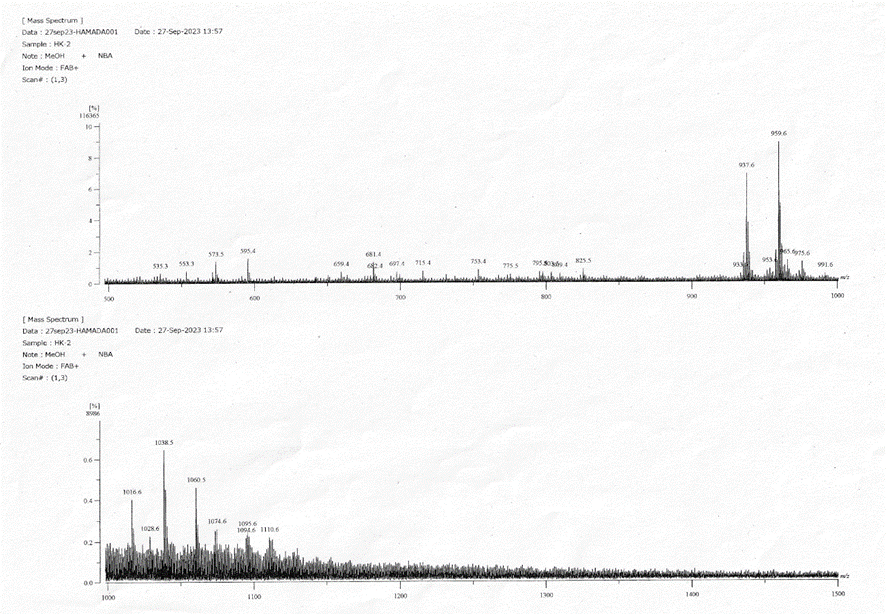
**

**Figure S9. FAB Mass spectrum of 2**

**
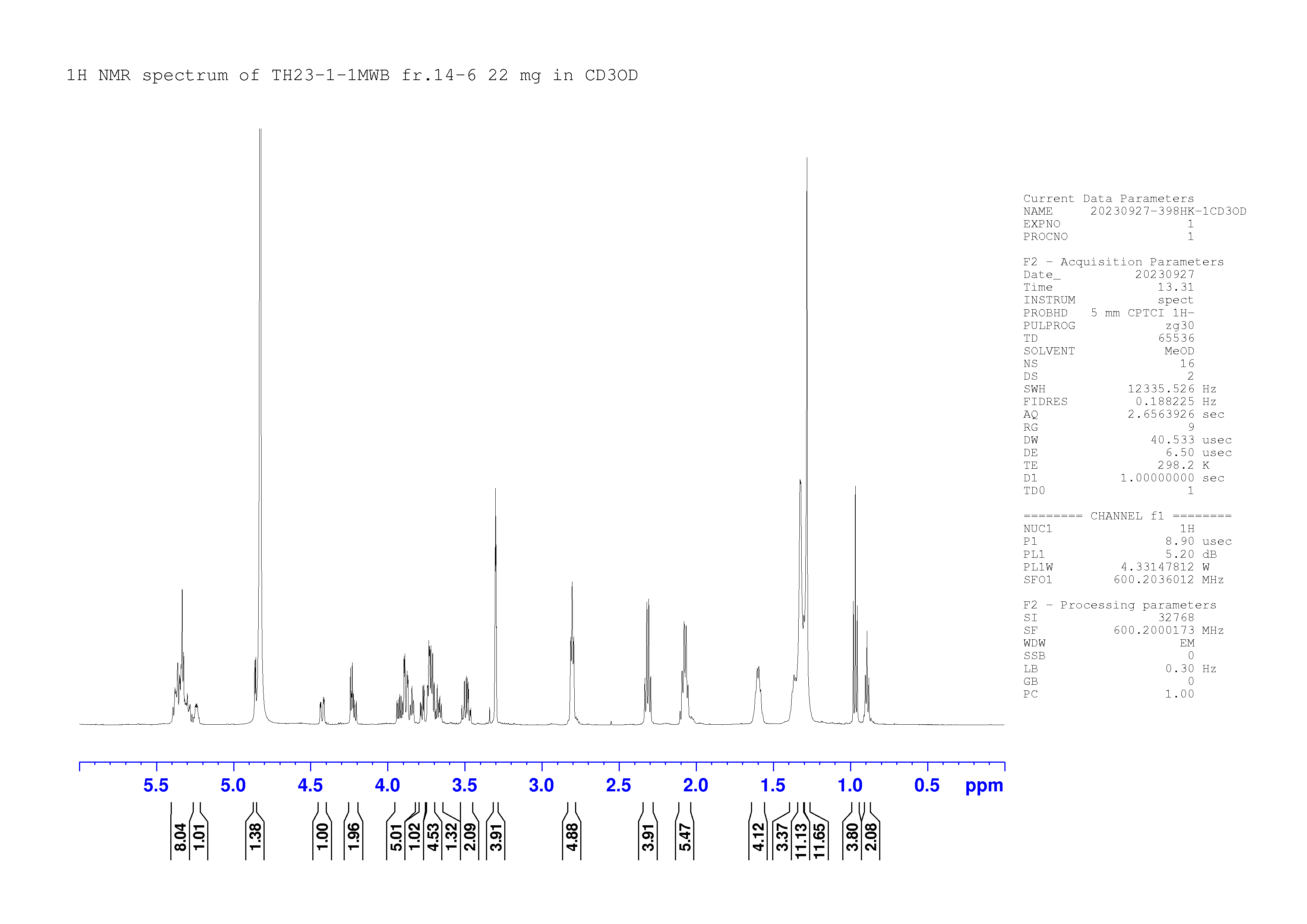
**

**Figure S10. ^1^H NMR spectrum of 2 in CD_3_OD (600 MHz / 300 K)**

**
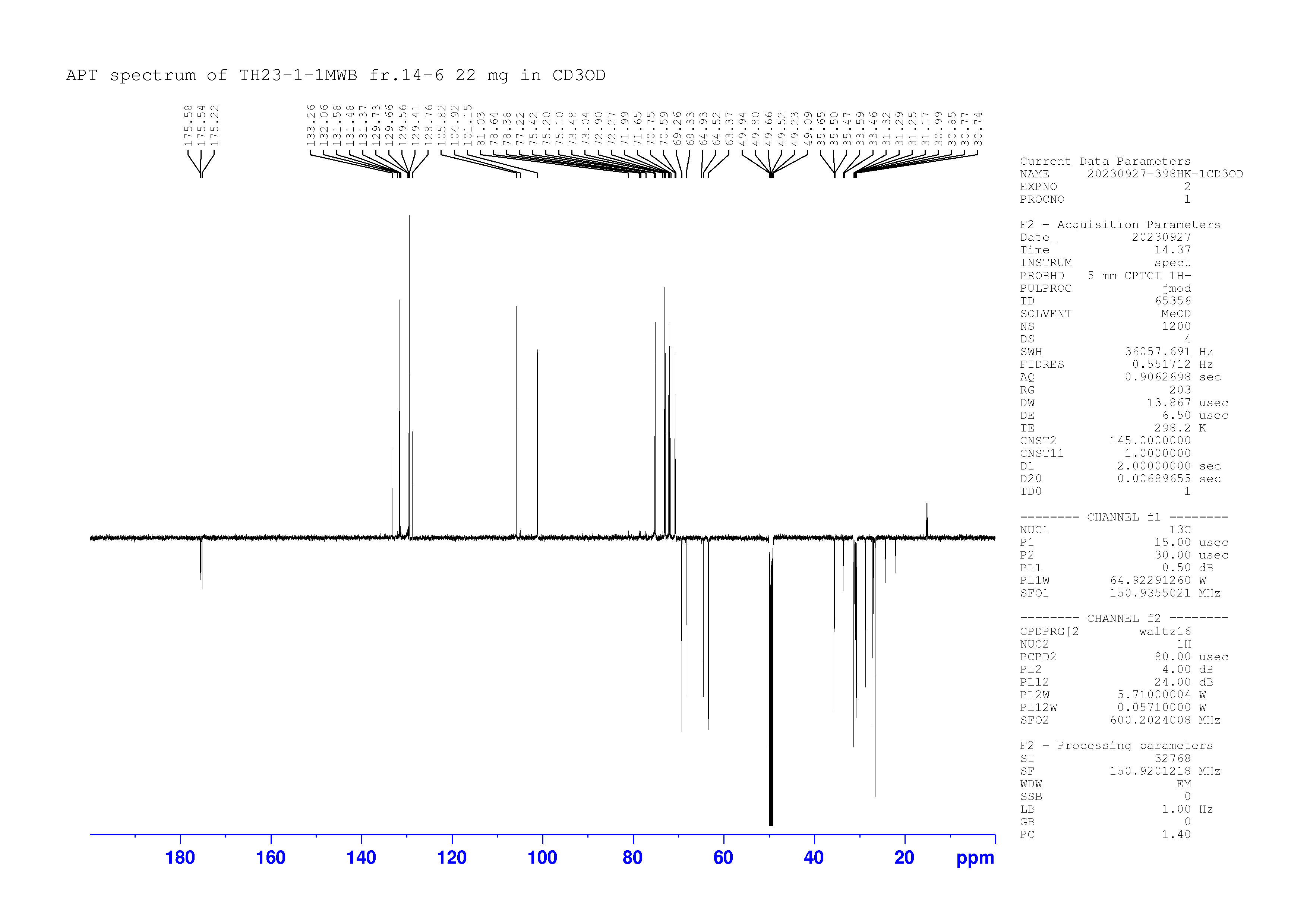
**

**Figure S11. APT spectrum of 2 in CD_3_OD (600 MHz / 300 K)**

**
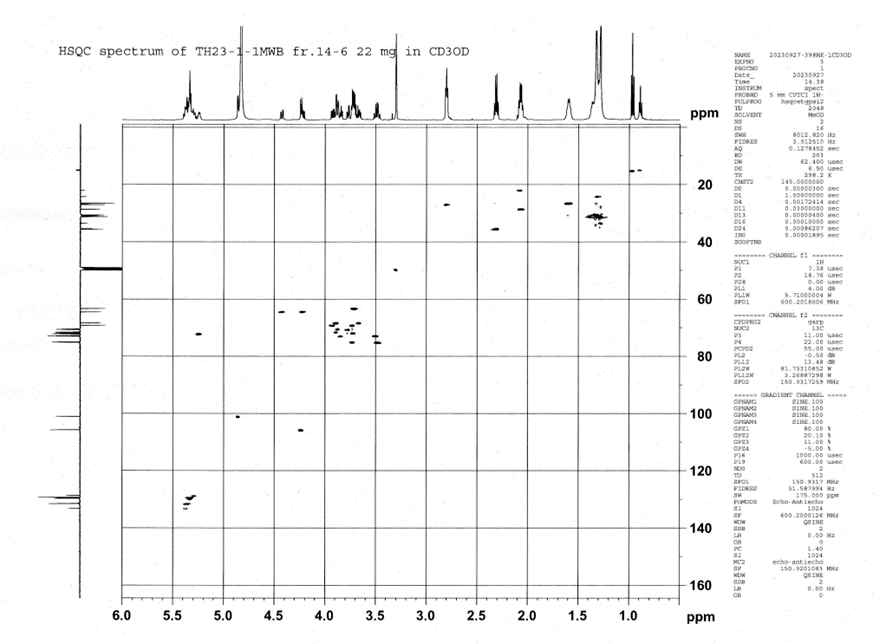
**

**Figure S12. HMQC spectrum of 2 in CD_3_OD (600 MHz / 300 K)**

**
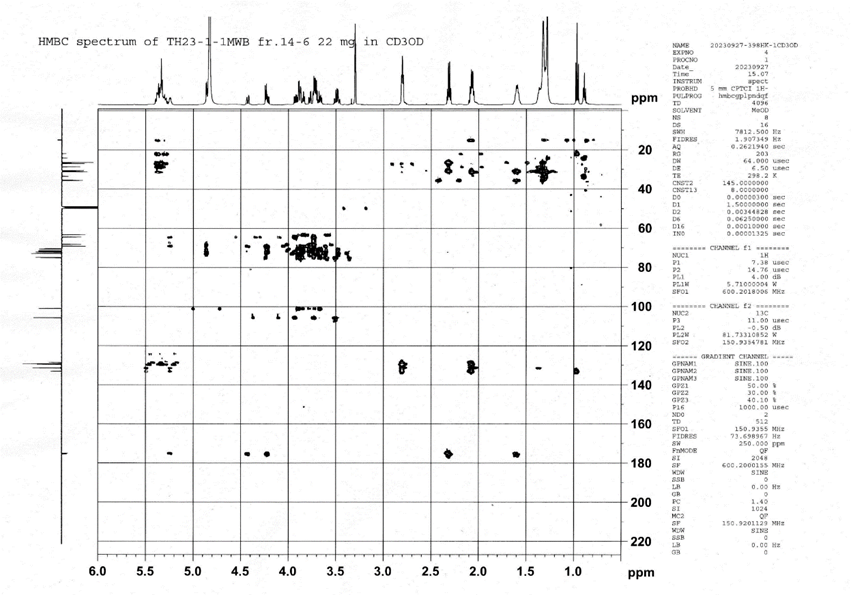
Figure S13. HMBC spectrum of 2 in CD_3_OD (600 MHz / 300 K)**

**
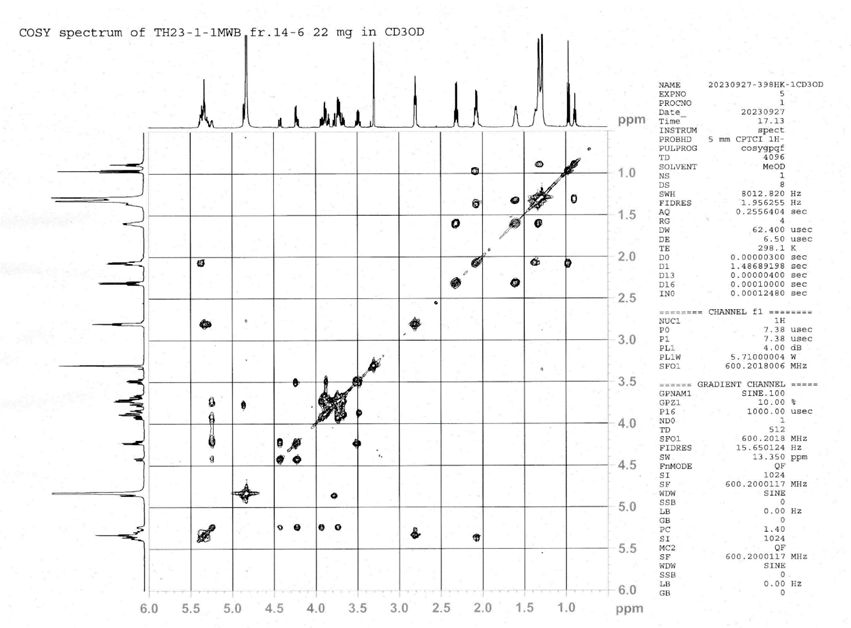
**

**Figure S14. ^1^H-^1^H COSY spectrum of 2 in CD_3_OD (600 MHz / 300 K)**

**
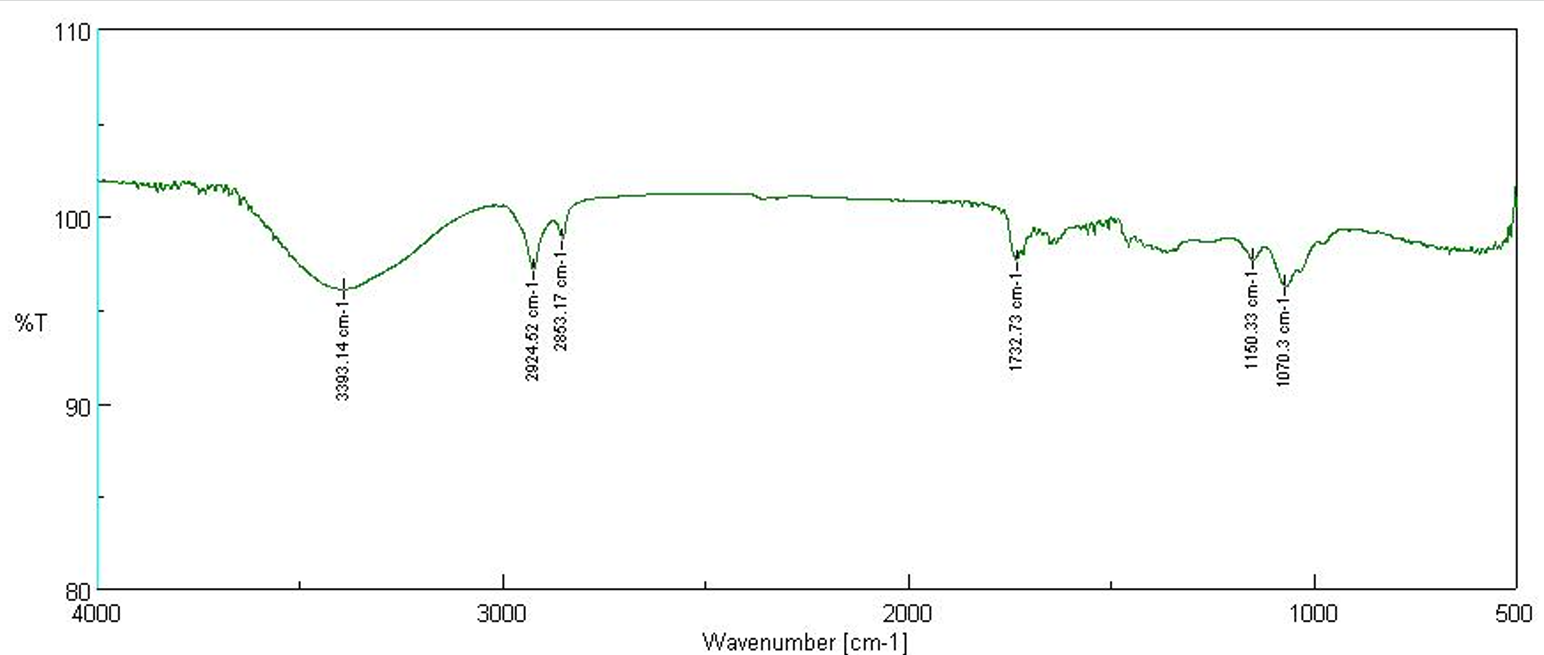
**

**Figure S15. IR spectrum of 2**

**
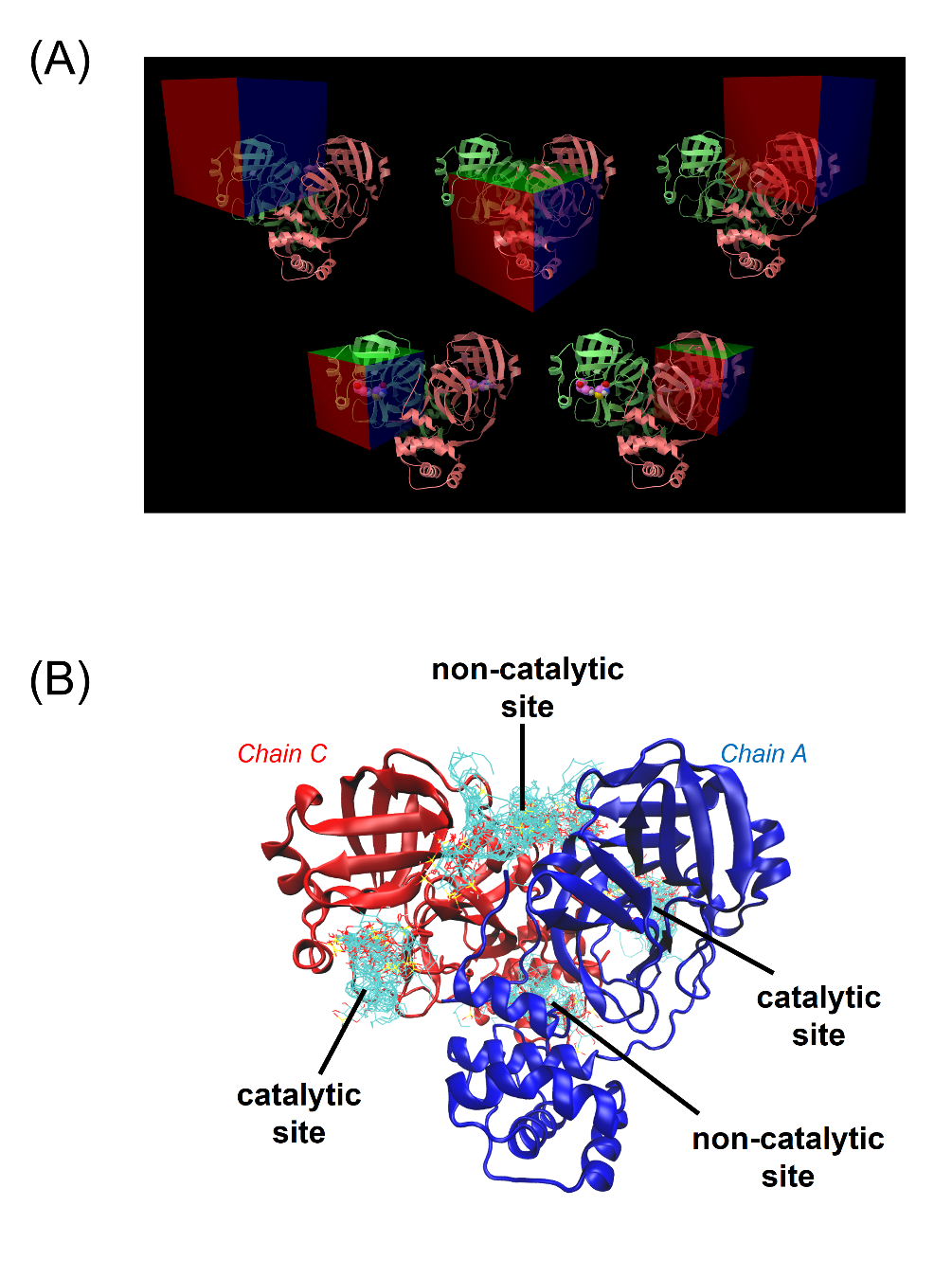
**

**Figure S16. Five target regions in M^pro^ dimer for docking calculations of SQDG (1)**
